# Iron-Imprinted Single-Atomic Site Catalyst-Based Nanoprobe for Detection of Hydrogen Peroxide in Living Cells

**DOI:** 10.1007/s40820-021-00661-z

**Published:** 2021-06-19

**Authors:** Zhaoyuan Lyu, Shichao Ding, Maoyu Wang, Xiaoqing Pan, Zhenxing Feng, Hangyu Tian, Chengzhou Zhu, Dan Du, Yuehe Lin

**Affiliations:** 1 School of Mechanical and Materials Engineering, Washington State University, Pullman, WA 99164 USA; 2 School of Chemical, Biological, and Environmental Engineering, Oregon State University, Corvallis, OR 97331 USA; 3 Irvine Materials Research Institute (IMRI), University of California, Irvine, CA 92697 USA

**Keywords:** Single-atomic site catalysts, Nanoprobe, Peroxidase-like activities, Biosensing, Living cell

## Abstract

Fe-based single-atomic site catalysts (SASCs), with the natural metalloproteases-like active site structure, have attracted widespread attention in biocatalysis and biosensing. Precisely, controlling the isolated single-atom Fe-N-C active site structure is crucial to improve the SASCs’ performance. In this work, we use a facile ion-imprinting method (IIM) to synthesize isolated Fe-N-C single-atomic site catalysts (IIM-Fe-SASC). With this method, the ion-imprinting process can precisely control ion at the atomic level and form numerous well-defined single-atomic Fe-N-C sites. The IIM-Fe-SASC shows better peroxidase-like activities than that of non-imprinted references. Due to its excellent properties, IIM-Fe-SASC is an ideal nanoprobe used in the colorimetric biosensing of hydrogen peroxide (H_2_O_2_). Using IIM-Fe-SASC as the nanoprobe, in situ detection of H_2_O_2_ generated from MDA-MB-231 cells has been successfully demonstrated with satisfactory sensitivity and specificity. This work opens a novel and easy route in designing advanced SASC and provides a sensitive tool for intracellular H_2_O_2_ detection.

**Supplementary Information:**

The online version contains supplementary material available at 10.1007/s40820-021-00661-z.

## Highlights

- A facile ion-imprinting method (IIM) is used to synthesize the isolated Fe-N-C single-atomic site catalyst (IIM-Fe-SASC), which mimics the natural enzyme-like active site and shows excellent peroxidase-like activity.
- The ion-imprinting process can precisely control ion at the atomic level and form numerous well-defined single-atomic Fe-N-C sites.
- The IIM-Fe-SASC has been successfully used as the nanoprobe for in situ H_2_O_2_ detection generated from MDA-MB-231 cells.

**Supplementary Information:**

The online version contains supplementary material available at 10.1007/s40820-021-00661-z.

## Graphical abstract

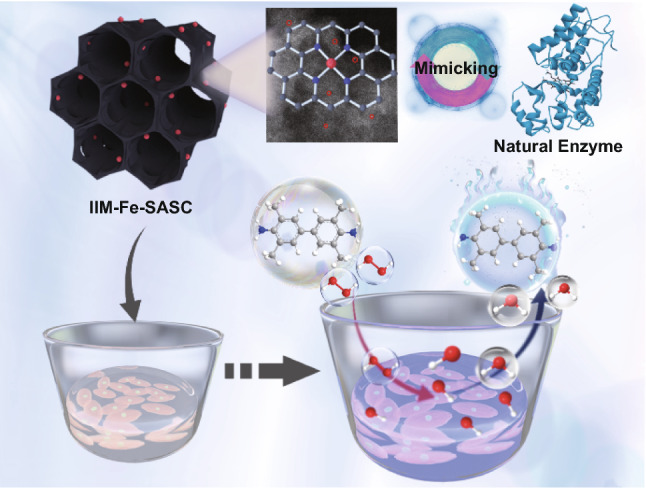

## Introduction

Hydrogen peroxide (H_2_O_2_), playing an important role in physiological processes and as a messenger molecule for cellular effects, is crucial for immune response and cell growth/senescence [1, 2] and also serve as potential biomarkers for diagnosis or monitoring diseases, such as diabetes, cancers, inflammation, cardiovascular and neurodegenerative diseases [3, 4]. Therefore, sensing H_2_O_2_ generated from living cells with high sensitivity and specificity will be of vital clinical value for disease diagnosis and better comprehension of disease mechanisms [5, 6]. However, due to the relatively low concentration of H_2_O_2_ in physiological environments, in vivo H_2_O_2_ sensing for clinical applications remains a challenge [7]. Developing highly sensitive H_2_O_2_-responsive probes has paramount importance for biosensing in living cells.

Single-atomic site catalysts (SASCs) containing atomically dispersed metal active sites demonstrate distinctive advantages in catalytic activities and selectivity for various catalytic reactions [8–14]. Recently, SASCs with peroxidase-like activity characteristics have attracted numerous attentions in the biosensing field due to their attractive properties of high stability and unprecedented catalytic performance toward H_2_O_2_ [15–18]. Therefore, Fe-based SASCs are considered as substitutes of natural horseradish peroxidase (HRP) owing to their maximum specific activity and atomic utilization and have been applied in biosensing and bioremediation [19–22]. For example, we reported a Fe-based SASC linked immunosorbent assay for early detection of Alzheimer’s disease, and an ultralow detection limit was achieved [23]. To rationally design Fe-based SASCs, researchers are usually devoted to selecting special precursors that either already contain single-atom metal species or use the coordination between the complex ligands and surface groups of support materials [24, 25]. Moreover, adsorbing iron ions to bulk materials or using a top-down synthetic method to peel off iron from metal bulk can also synthesize SASCs [26–28]. These methods have drawbacks of using expensive organic macromolecule complexes and running the risk of aggregating single-atom metal species into nanosized metal counterparts [29, 30]. The resulted SASCs either require high cost or possess a relatively low density of the active sites, which limit their large-scale practical applications. Therefore, new strategies for constructing coordination sites for preparing Fe-based SASCs are urgently needed.

Ion-imprinting technology (IIT) is a type of molecularly imprinted technology that involves self-assembly of the interested ion (the template), complementary functional monomers and cross-linkers to synthesize imprinted materials [31–33]. Generally, pre-polymerized complex systems can be formed during the preassembled system with each isolated template ion interacting with function monomer independently, and the template ions are embedded and isolated in the cross-linked matrix after the polymerization process. Due to the pro-coordination process between ions and functional monomers, the activate sites are precisely controlled at the atomic level and high-density single-atom irons are obtained. Hence, based on the advantages of IIT, it is believed that utilizing IIT can effectively confine the ion in the matrix and achieve a high yield of SASCs with a low-cost and straightforward process [34].

**Fig. 1:**
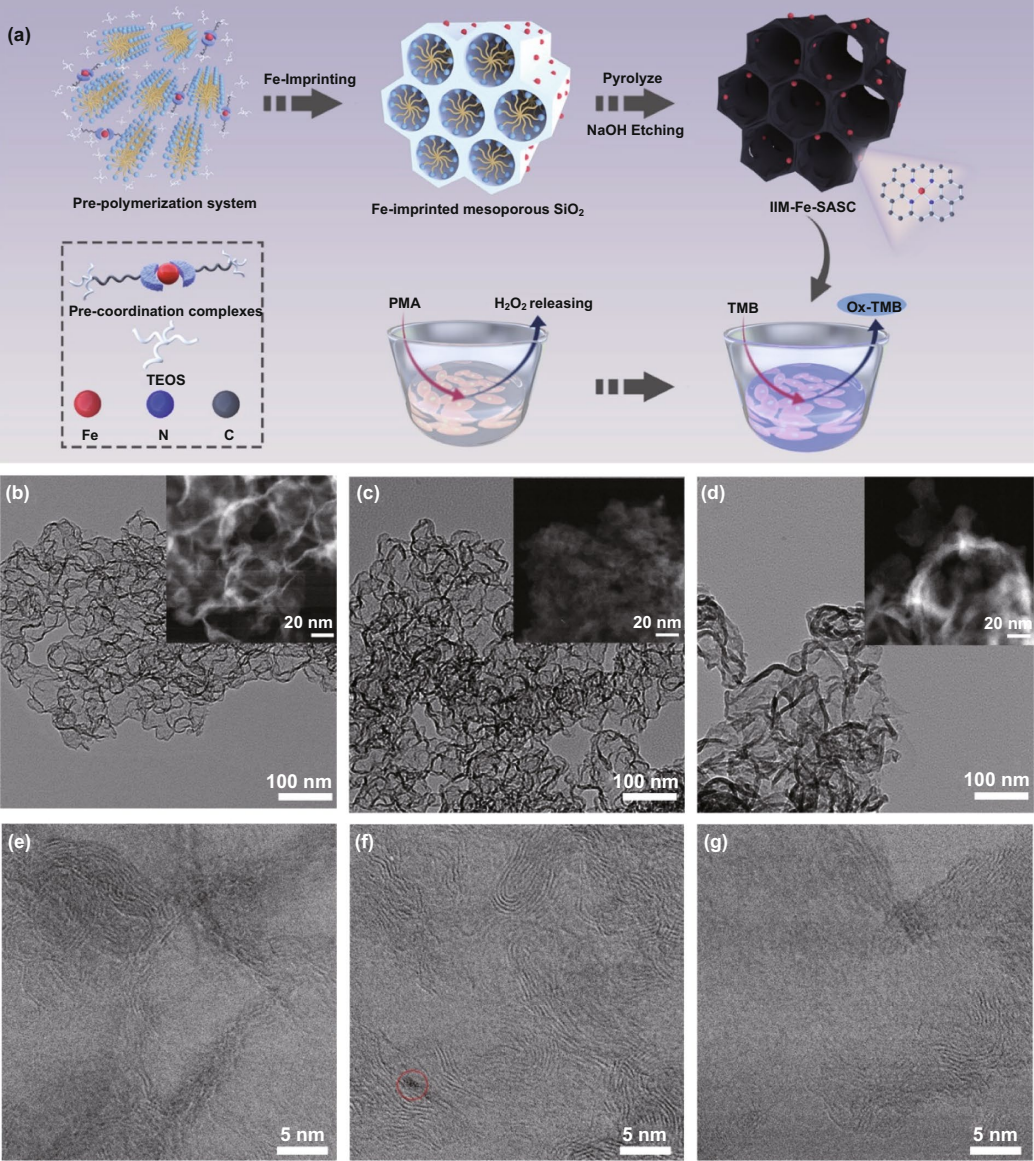
**a** Schematic diagram of the process steps for IIM-Fe-SASC nanoprobe synthesis and in situ intracellular hydrogen peroxide detection. **b–d** TEM images of IIM-Fe-SASC, NIM-Fe-SASC, and NIM in different resolutions. **e–g** STEM bright-field images for IIM-Fe-SASC, NIM-Fe-SASC, and NIM at higher magnification

Hence, a facile ion-imprinting approach was used to synthesize the Fe-based single-atomic site catalyst (IIM-Fe-SASC) [35], and the developed Fe-SASC was used as a nanoprobe for in situ intracellular H_2_O_2_ detections (Fig. 1a). For synthesizing the IIM-Fe-SASC, the mesoporous silica was used as the matrix in the imprinted materials to prevent aggregation of the isolated iron ions. The [3-(2-Aminoethylamino)propyl]trimethoxysilane (A-Tri-EOS) was selected as functional monomers for that it could provide coordination sites to immobilize the iron atom. Precisely, controlled high-density single-atomic activate sites were achieved during the pro-coordination process between iron ions and A-Tri-EOS. IIM-Fe-SASC with inherent peroxidase-like activity could catalyze H_2_O_2_ to reactive oxygen species. In this paper, we successfully applied IIM-Fe-SASC as the sensing probe in a typical colorimetric assay to detect H_2_O_2_ with ultrahigh sensitivity and specificity. The IIM-Fe-SASC showed better peroxidase-like ability than that of non-imprinted references. Importantly, in situ detection of H_2_O_2_ generated from breast cancer cells (MDA-MB-231) was performed using the IIM-Fe-SASC-based assay, which demonstrates the practical clinic applications of SASC nanoprobe.

## Experimental

### Preparation of Single-Atomic Site Catalyst Nanoprobe

To prepare IIM-Fe-SASC, the mesoporous structure-directing micelles (solution 1) was first prepared by adding 0.6 g cetyltrimethylammonium bromide (CTAB) in a NaOH solution under vigorous stirring. 11 mg ion template Fe (NO_3_)_3_ and 0.25 mL functional monomers A-Tri-EOS were dissolved together and shaken for 15 min (solution 2), aiming to form the pre-coordination complexes of iron ions-functional monomers. Then, solution 2 and 2.5 mL cross-linking agent tetraethyl orthosilicate (TEOS) were added dropwise to solution 1, and the Fe-imprinted mesoporous SiO_2_ could be obtained through filtration after reaction for 2 h. Meanwhile, to analyze and compare the performance of IIM-Fe-SASC, two more materials were synthesized and used as compare samples: Fe-based SASC synthesized using an adsorbing method (NIM-Fe-SASC) and a non-imprinted nanomaterial without Fe ions (NIM). For the adsorbing method, non-imprinted mesoporous SiO_2_ was prepared with the same route without adding iron ions. Subsequently, the obtained mesoporous SiO_2_ was used to absorb the same amount of Fe(NO_3_)_3_ solution and then the Fe-adsorbed mesoporous SiO_2_ was collected after filtration. The Fe-imprinted mesoporous SiO_2_, Fe-adsorbed mesoporous SiO_2_ and mesoporous SiO_2_ were pyrolyzed at 900 °C for 30 min in N_2_ atmosphere and then 30 min in NH_3_ atmosphere. Then, the isolation matrix SiO_2_ was removed by soaking in 3 M NaOH at 80 °C for 48 h. The synthesized samples are named IIM-Fe-SASC (by imprinting method), NIM-Fe-SASC (by adsorbing method) and NIM (without adding Fe iron), respectively. Further, specific evaluating peroxidase-like activity processes are described in Supplementary Information.

### Detect H_2_O_2_ Produced from the Breast Cancer Cells

In order to evaluate the H_2_O_2_ released from MDA-MB-231 cells, the cells were placed in a 96-well plate for 24 h. After that, the plates were washed three times using PBS solution. Then, PMA solution (20 μL, 2 μM) and 100 μL PBS were sequentially added and incubated for 30 min. Finally, IIM-Fe-SASC (10 mg mL^−1^, 50 μL), TMB (1 mM, 100 μL) and HAC-NaAC buffer (100 μL, pH 4.0) were subsequently added and incubated for 5 min. Finally, a multi-mode reader was used to record the absorbance at 652 nm.

### Intracellular Imaging

For live/dead cell imaging, MDA-MB-231 cells were seeded in 6-well plates and incubated overnight at 37 °C in a cell culture incubator. Add IIM-Fe-SASC nanoprobe or TMB with 10 mg mL^−1^ and 1 mM and incubate in the dark for 30 min, respectively. Subsequently, the calcein-AM (2 µM) and EthD-1 (4 µM) solutions from the live/dead viability kit were added to each well and incubated for 15 min. Finally, after washing thoroughly with PBS, observe the cells with CLSM (Leica TCS SP8). Here, the green fluorescence from Calcein-AM represents living cells, and the red fluorescence from EthD-1 represents dead cells. For the intracellular ROS imaging, DCFH-DA was used to stain cells as a ROS fluorescent probe. MDA-MB-231 cells were seeded in 12-well plates and incubated for 24 h in a cell culture incubator containing 5% CO_2_ and 95% humidity. Then, the cells were incubated with IIM-Fe-SASC nanoprobe for 4 h under 0, 2.5, 5 and 10 μg mL^−1^, respectively. Herein, the IIM-Fe-SASC nanoprobe was broken down to nanosize via an intense ultrasound treatment for use in endocytosis. Then, 10 μM DCFH-DA was added to each well, followed by incubation for 20 min. The cells were washed using PBS. Finally, fluorescence images were obtained by CLSM.

## Results and Discussion

### Materials Characterizations

Transmission electron microscopy (TEM) was used to study the structures and morphologies of as-prepared samples. Figure S1 shows well-defined mesoporous structures in both Fe-imprinted and non-Fe-imprinted mesoporous SiO_2_ precursors. No obvious structural difference between them is found, indicating that the sol–gel polymerization and mesoporous SiO_2_ precursor structures are not affected when adding Fe ions. The obtained IIM-Fe-SASC, NIM-Fe-SASC and NIM show the inhomogeneous structure (Fig. 1b–d). Moreover, in Fig. 1e–g, distorted graphite layers were observed by scanning TEM (STEM), which makes the catalysts rich in defects and nanopores, thus accommodates a large amount of single-atom active sites. The STEM images of IIM-Fe-SASC are the same as that of NIM, where no nanoparticles are observed, suggesting that iron atoms embed into the IIM-Fe-SASC as dispersive isolated atoms. However, nanocrystal can be found in NIM-Fe-SASC and is marked in the red circle in Fig. 1f, illustrating that the adsorption method can easily produce metal clusters and are hard to remove. X-ray diffraction (XRD) pattern demonstrates that the IIM-Fe-SASC possesses nanoparticle-free features (Fig. S2).

**Fig. 2:**
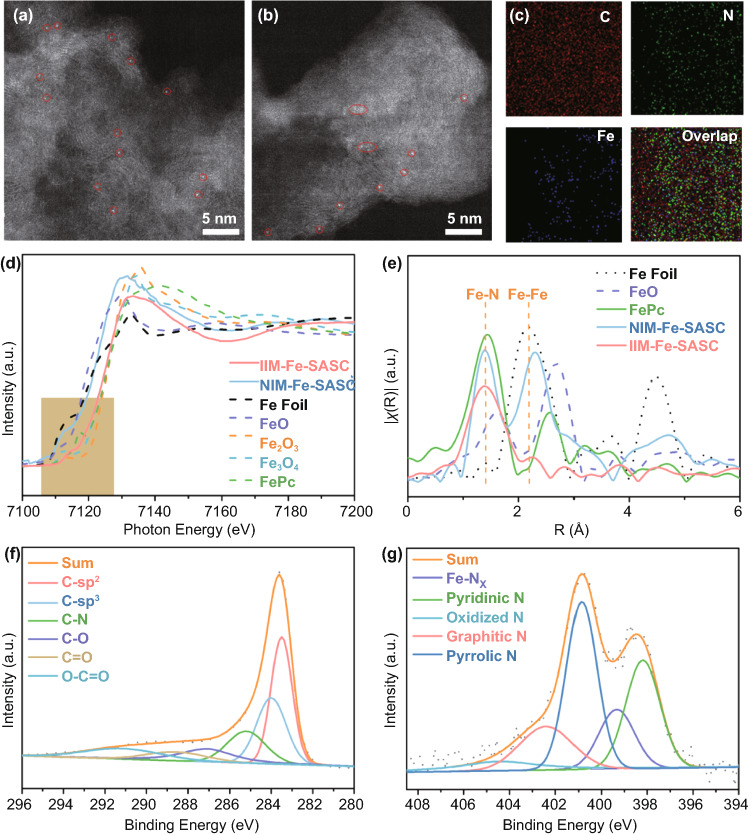
**a**, **b** HAADF-STEM images of IIM-Fe-SASC and NIM-Fe-SASC, respectively. **c** Elemental -mapping images of IIM-Fe-SASC. **d** Fe K-edge XANES spectra of IIM-Fe-SASC, and reference samples of FePc, Fe foil, FeO, Fe_2_O_3,_ and Fe_3_O_4_. **e** FT *k*^2^-weighted EXAFS R-space spectra of IIM-Fe-SASC, NIM-Fe-SASC, FePc, Fe foil, and Fe_2_O_3_. **f**, **g** C 1*s* and N 1*s* XPS spectra of IIM-Fe-SASC

To further prove the state of single iron atom, aberration-corrected high-angle annular dark-field STEM (HAADF-STEM) was employed to investigate the wall structure of the as-made IIM-Fe-SASC and NIM-Fe-SASC at the atomic level. For IIM-Fe-SASC, as marked in red circles in Fig. 2a, uniformly dispersed single-atom Fe sites show on the carbon matrix. Nevertheless, NIM-Fe-SASC, prepared by the traditional adsorption method, has both single iron atoms and some stacked metal crystals (Fig. 2b), further demonstrating that the doped Fe species are not purely single atoms. Elemental composition and distribution in IIM-Fe-SASC were detected by auxiliary energy-dispersive X-ray spectroscopy (EDS) elemental analysis. Figure 2c shows the corresponding element maps of carbon, nitrogen and iron in IIM-Fe-SASC. All elements are uniformly distributed in the IIM-Fe-SASC, indicating that nitrogen coordinated with Fe atoms can be doped into the carbon matrix. The Fe atom loading is confirmed as 2.12 wt%, which was measured by inductively coupled plasma mass spectrometry (ICP-MS).

X-ray absorption spectroscopy (XAS) measurements [36, 37] were performed to determine the local structural and electronic states of Fe atoms in the as-synthesized catalysts. The absorption edge of Fe K-edge X-ray absorption near edge structure (XANES) spectrum of IIM-Fe-SASC is located at higher energy compared with that of Fe foil, but in between two reference samples (FeO and Fe_2_O_3_), indicating that the Fe atoms in IIM-Fe-SASC have a positive charge (Fig. 2d) that is between + 2 and + 3. Note that, the absorption edge of IIM-Fe-SASC almost overlaps with that of FePc, which may indicate the similarity of their local structure surrounding Fe atoms. Moreover, in Fig. 2e, the Fourier-transformed (FT) *k*^2^-weighted EXAFS curve of IIM-Fe-SASC only shows a main peak at about 1.4 Å, which is aligned with the Fe–N peak in the FePc reference sample, suggesting that Fe is in single‐atom dispersed form with Fe–N bonding. In comparison, besides the Fe–N scattering path, a Fe–Fe peak at 2.2 Å (comparing with Fe foil) exists in NIM-Fe-SASC, demonstrating Fe exists as both Fe–N_*x*_ motifs and metallic Fe clusters. These results are consistent with the HAADF-STEM results shown above. It is also noted that the *k*-space EXAFS oscillations (Fig. S3) reveal that IIM-Fe-SASC spectrum is different from those of Fe foil and Fe oxides, but similar to that of Fe single-atom reference FePc, which is mainly due to the fact that the selected functional monomers effectively coordinate iron atoms during the ion-imprinting process [24]. The chemical composition of the obtained IIM-Fe-SASC was conducted by X-ray photoelectron spectroscopy (XPS). The high-resolution C 1*s* spectrum of the IIM-Fe-SASC (Fig. 2f) can be deconvoluted into four components of C-*sp*^2^ (283.6 eV), C-*sp*^3^ (284.0 eV), C–N (285.2 eV), C–O (287.1 eV), C=O (288.6 eV) and O–C=O (289.1 eV) [38–40]. The ratio of C-*sp*^2^ in IIM-Fe-SASC is 33.4%, much lower than the reported high graphitized carbon materials (like graphene) [41, 42], indicating that the IIM-Fe-SASC has a lower degree of graphitization and abundant defects and edges. Raman spectra were also used to study graphitizing degrees (Fig. S4). A strong D band and the relatively high-intensity ratio of D band to G band (~ 0.91) further demonstrate the numerous existed defects and structural imperfections of IIM-Fe-SASC [43]. The complex N 1*s* spectrum of IIM-Fe-SASC is deconvoluted into several main peaks (Fig. 2g), which correspond to pyridinic N (398.2 eV), pyrrolic N (400.9 eV), graphitic N (402.4 eV) and oxidized N (404.7 eV), respectively. [24, 44] Most important, a spectral valley between two dominating pyridinic peak and pyrrolic peak at 399.3 eV indicates the presence of Fe–N_*x*_ single-atom sites, [45] which is in good agreement with the result of EXAFS. Besides, the Fe 2*p* spectra are shown in Fig. S5, which further illustrates the successful Fe doping.

### Peroxidase-like Activities Evaluation

**Fig. 3:**
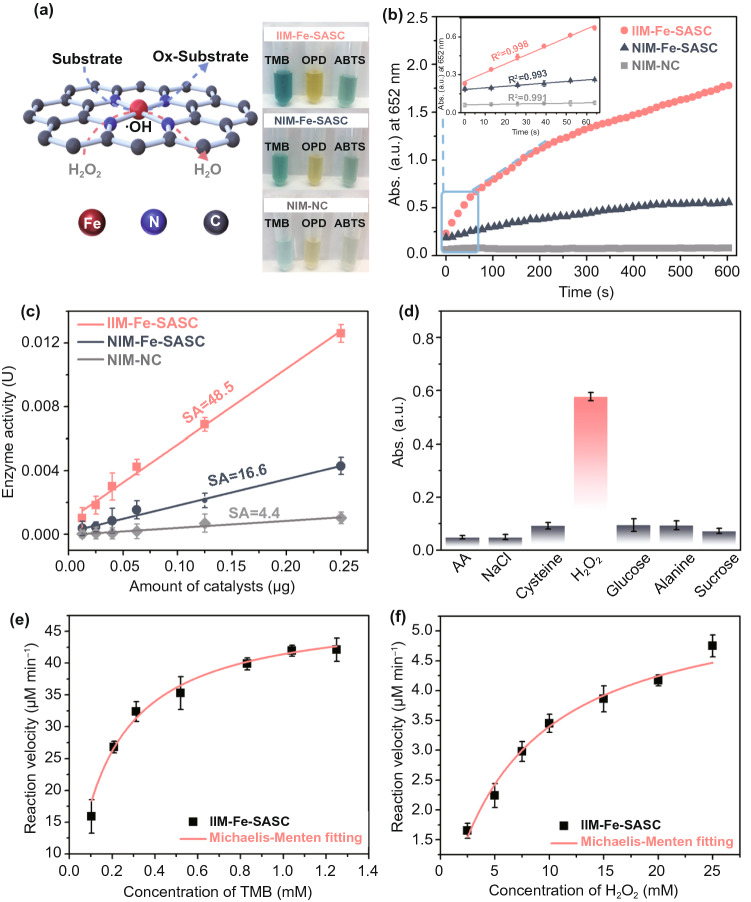
**a** Mechanism illustration of IIM-Fe-SASC catalyzed and chromogenic reaction with different substrates. **b** Absorbance-time curves and magnified initial linear portion of TMB chromogenic reaction catalyzed by IIM-Fe-SASC, NIM-Fe-SASC, and NIM. **c** Specific activities of IIM-Fe-SASC, NIM-Fe-SASC, and NIM. **d** Specificity evaluation of IIM-Fe-SASC for various interferences. **e–f** Steady-state kinetics curves of IIM-Fe-SASC toward TMB and H_2_O_2_

The peroxidase-like activities of the IIM-Fe-SASC, NIM-Fe-SASC and NIM are verified, and the results of the chromogenic reaction are shown in Fig. 3a. The obvious color change of IIM-Fe-SASC can be observed and is caused by the oxidation of colorless substrates to their corresponding oxidized products. Notably, NIM cannot trigger any chromogenic reaction regardless of the existence of H_2_O_2_, which proves that the peroxidase-like property of IIM-Fe-SASC is mainly originated from Fe–N_*x*_ sites. The peroxidase-like activity of IIM-Fe-SASC and control samples were conducted, the results are shown in Fig. 3b. Absorbance at 652 nm increases along with reaction time, and linear relations with *R*^2^ coefficient close to 1 are obtained by linear regression analysis during the first minute. It is clear that IIM-Fe-SASC has the best peroxidase-like catalytic performance. Then, the catalytic activities expressed in units (U) of IIM-Fe-SASC, NIM-Fe-SASC and NIM were further evaluated (Fig. 3c). The peroxidase-mimic activity of IIM-Fe-SASC is calculated to be 48.5 U mg^−1^, which is much higher than that of NIM-Fe-SASC (16.6 U mg^−1^) and NIM (4.4 U mg^−1^) and also superior to most of the reported peroxidase-mimic nanomaterials (Table S1). Herein, the added Fe amount in the precursor was also optimized. As shown in Table S2, the obtained IIM-Fe-SASC by adding 10 mg Fe precursor own the best enzyme-like activity. As the Fe precursor increases from 5 to 10 mg, the single-atom iron will also be increased, boosting the enzyme-like activity. However, adding an excessive amount of Fe precursor exceeds the maximum confinement capability of SiO_x_ matrix, resulting in forming some iron crystal during pyrolysis process (Fig. S6). The peroxidase-mimic activity of IIM-Fe-SASC is more than tenfold than that of NIM, which further illustrates that the activity is derived from Fe–N_*x*_ active sites. What’s more, the huge activity gap between IIM-Fe-SASC and NIM-Fe-SASC proves that applying IIT results in relatively high-density atomic Fe-N_x_ active sites, thus boosting the peroxidase-like performance. For comparison, the specific activity of natural HRP is evaluated to be 263.8 mg mL^−1^ under the same process, which is in accordance with the manufacture’s value (≥ 250 U mg^−1^), and the specific activity of the IIM-Fe-SASC is approaching that of natural HRP.

By comparing the detection performances of H_2_O_2_ (100 mM) and its interfering substrates (500 mM) of cysteine, ascorbic acid, NaCl, glucose, alanine and sucrose, the selectivity of IIM-Fe-SASC toward H_2_O_2_ was revealed and shown in Fig. 3d, indicating IIM-Fe-SASC has a satisfactory selectivity toward H_2_O_2_. Furthermore, the steady-state kinetics curves of IIM-Fe-SASC toward H_2_O_2_ and TMB substrates were obtained and shown in Fig. 3e, f, while HRP was used to serve as a reference for comparison (Fig. S7). Typical Michaelis–Menten curves and the double reciprocal plots of initial reaction rates (Fig. S8) are observed. By fitting in Michaelis–Menten model, Michaelis–Menten parameters of IIM-Fe-SASC and nature HRP toward TMB and H_2_O_2_ are obtained (Table S3). IIM-Fe-SASC shows a comparable *K*_m_ toward H_2_O_2_ compared with natural HRP, and *K*_m_ of IIM-Fe-SASC to TMB is lower than that of natural HRP, indicating the IIM-Fe-SASC has a higher affinity toward TMB and a similar affinity level toward H_2_O_2_. We further analyzed the potential effects of harsh environments of temperature and pH on the peroxidase-like activity of IIM-Fe-SASC. As shown in Fig. S9, IIM-Fe-SASC can preserve their activity in a wide pH range of 2.5–8.5, while maintained above 80% activity from 4 to 80 °C, which shows satisfaction robustness against the harsh environment.

### Mechanisms for Peroxidase-like Activity

**Fig. 4:**
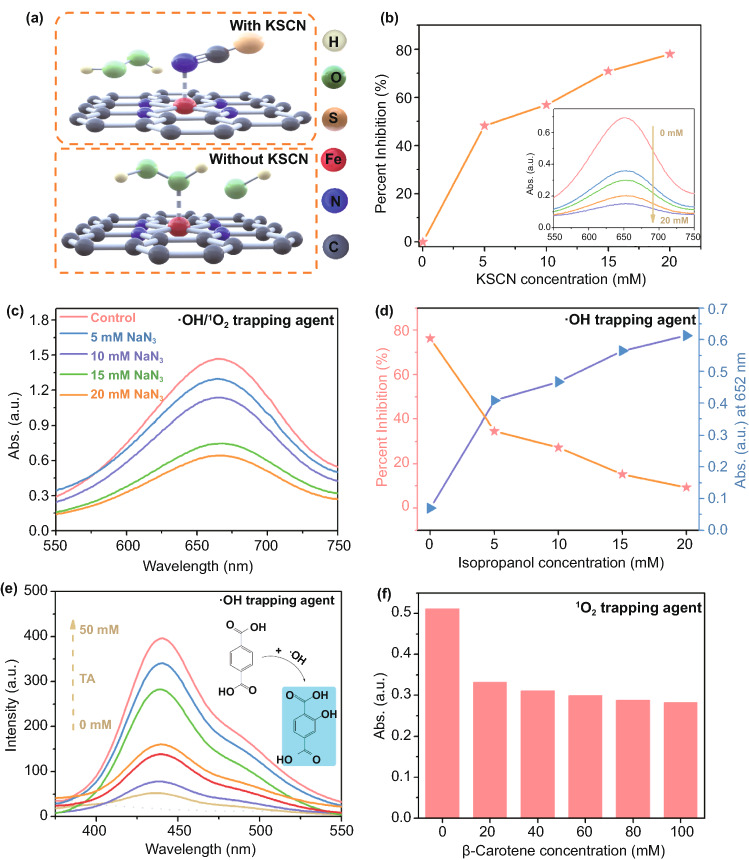
**a** Schematic illustration of the mechanism of KSCN influence. **b** Percent inhibition and absorption change of IIM-Fe-SASC + TMB + H_2_O_2_ solution upon the addition of various concentrations of KSCN. **c** Absorbance changes after adding various amounts of NaN_3_. **d** Percent inhibition and absorption change after adding isopropanol. **e** TA as a •OH fluorescent probe. **f** Absorption change after adding β-carotene with different concentrations

Thiocyanate ions (SCN^−^) were used to evaluate the role of single-atom Fe in catalytic efficiency because SCN^−^ and Fe-centered catalytic sites can form a stable chelate complex, thereby block Fe activity sites and fail to decompose H_2_O_2_. The mechanism illustration is shown in Fig. 4a [46]. As shown in Fig. 4b, the inhibitory effect of peroxidase-like activity is significantly enhanced with the increase in SCN^−^. These results further prove that the peroxidase-like activity of Fe-SASC is mainly generated from the atomically dispersed Fe–N_*x*_ active sites, which is consistent with the huge specific activity difference in Fig. 3c. The active intermediates were also investigated using various scavengers (Fig. 4c–f). In Fig. 4c, the absorbance value of ox-TMB decreases significantly with the addition of NaN_3_, indicating that the participation of •OH/^1^O_2_ is related to the oxidation coloration reaction [47]. The generated •OH was detected by the enhanced isopropanol inhibition ability (Fig. 4d). The higher fluorescent signal of terephthalic acid (TA) catalyzed by IIM-Fe-SASC nanoprobe demonstrated that more •OH is generated (Fig. 4e) [48, 49]. Besides, experimental results related to β-carotene verified the little presence of ^1^O_2_ (Fig. 4f) [50].

### Hydrogen Peroxide Detection in Living Cells

**Fig. 5:**
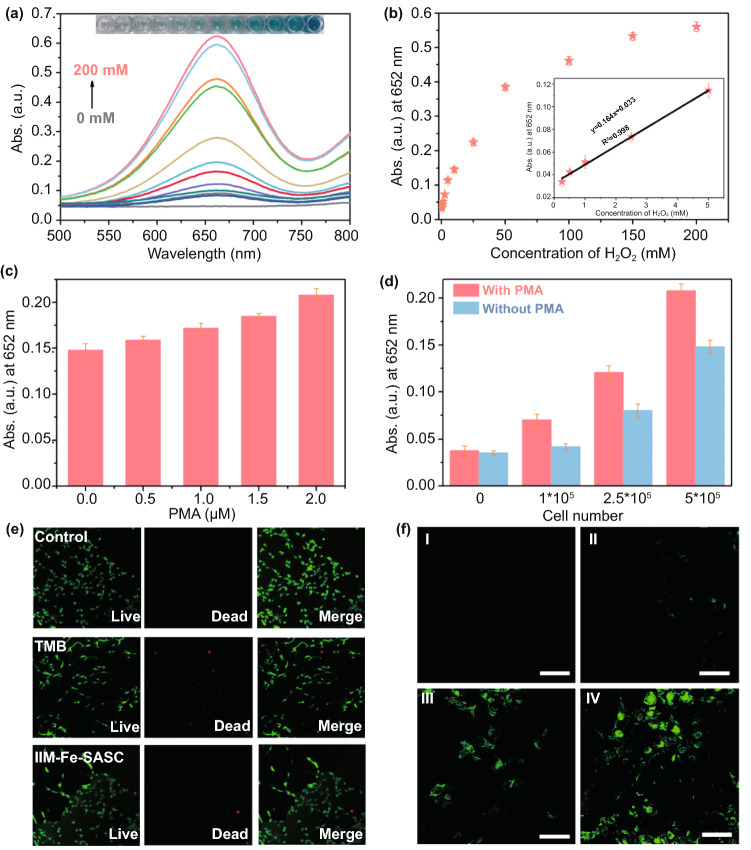
**a** UV–vis spectra of TMB oxidized by the IIM-Fe-SASC under different H_2_O_2_ concentrations. **b** Absorbance changes and linear calibration plot (inset) for H_2_O_2_ detection. **c** Absorbance values of H_2_O_2_ generated from MDA-MB-231 cells after treating with various concentrations of PMA. **d** Absorbance comparison toward MDA and PMA-treated MDA-MB-231 cells. **e** Fluorescence imaging of MDA-MB-231 cancer cells cultured with TMB and IIM-Fe-SASC nanoprobe. **f** CLSM images of active intermediates generated in MDA-MB-231 cells treated with different amount of IIM-Fe-SASC nanoprobe (I: 0 μg mL^−1^; II: 2.5 μg mL^−1^; III: 5 μg mL^−1^; IV: 10 μg mL^−1^). The intracellular ROS generation was detected by the DCFH-DA (Scale bar: 60 μm)

The linear detection range of IIM-Fe-SASC nanoprobe to H_2_O_2_ is determined and the results are shown in Fig. 5a. Accordingly, a fine linear relationship of H_2_O_2_ concentration to absorbance curve is achieved in the range of 0.25–5 mM (Fig. 5b). The MDA-MB-231 breast cancer cells were used for intracellular hydrogen peroxide detection. First, a standard MTT assay was carried out to verify the potential toxicity of TMB and IIM-Fe-SASC (Fig. S10). It is clear that the added TMB has little effect on cell viability. Furthermore, after 24 h of incubation in IIM-Fe-SASC with a concentration range of 1.0–10 μg mL^−1^, MDA-MB-231 cells can still retain their high viability, revealing the excellent biocompatibility of IIM-Fe-SASC. Adenosine-5-diphosphate (ADP), *N*-formylmethionyl-leucyl-phenylalanine (fMLP) and phorbol-12-myristate-13-acetate (PMA) were used to stimulate MDA-MB-231 cells, and the released H_2_O_2_ was detected (Fig. S11), in which PMA exhibited the optimal stimuli [51]. Then, PMA was then selected to treat MDA-MB-231 cells under different concentrations. The results in Fig. 5c show that the absorbance value is highly dependent on PMA concentrations. Furthermore, different cell numbers were treated with or without PMA. A higher colorimetric response is observed as the cell number increased, which can be ascribed to more H_2_O_2_ produced during PMA stimulation (Fig. 5d). Also, according to the H_2_O_2_ detection calibration curve in Fig. 5b, H_2_O_2_ concentration produced from the MDA-MB-231 cells (2.5 × 10^5^ cells/plate) is calculated to be 0.535 mM, and the average molecule number of H_2_O_2_ released in one cell (*N*_0_) is 3.48 × 10^11^ (calculated by the Avogadro equation: *n* = *N*_0_/*N*_A_, in which Avogadro’s constant *N*_A_ is 6.02 × 10^23^ mol^−1^). This value is in good agreement with the previous reports [49, 52], indicating that the colorimetric detection method based on IIM-Fe-SASC nanoprobe can be used in practical clinic applications.

A standard staining method was also used to evaluate the biocompatibility by a live/dead viability kit. The calcein-AM can combine with the living cell through the cell membrane and green fluorescence can be observed in the cytoplasm through a fluorescent microscope. As shown in Fig. 5e, MDA-MB-231 cells were cultured with IIM-Fe-SASC nanoprobe and TMB under testing concentrations. The CLSM images show that the TMB has minor effects on cell viability. And for IIM-Fe-SASC nanoprobe, no significant cell viability changes. Intracellular H_2_O_2_ detection was also performed through transporting IIM-Fe-SASC nanoprobe into MDA-MB-231 cells by endocytosis. Since we have already proved that the •OH and ^1^O_2_ are active intermediates during the peroxidase-like catalytic reaction, these intracellular reactive oxygen species can be evaluated using a fluorescence probe 2′,7′-dichlorofluorescein diacetate (DCFH-DA) [53]. As illustrated in Fig. 5f, MDA-MB-231 cells show insignificant green fluorescence when incubating with IIM-Fe-SASC nanoprobe. In contrast, obvious green fluorescence is observed in control cells (Fig. 5f–I), suggesting IIM-Fe-SASC nanoprobe can produce massive intracellular active intermediates. In addition, the intensity of the green fluorescence signal is also enhanced with the increasing concentration of nanoprobes. These results further demonstrate the excellent intracellular H_2_O_2_ detection ability of IIM-Fe-SASC nanoprobe.

## Conclusion

In summary, we have used a facile ion-imprinting approach to synthesize a Fe-based single-atom nanoprobe for hydrogen peroxide detection in living cells. The resultant IIM-Fe-SASC shows better peroxidase-like activity than that of non-imprinted references, demonstrating that the ion-imprinting process can precisely control ion at the atomic level and form numerous well-defined single-atom iron. High sensitivity and specificity of IIM-Fe-SASC nanoprobe have been achieved for colorimetric detection of H_2_O_2_. Furthermore, in situ detection of H_2_O_2_ generated from the MDA-MB-231 cells was performed, exhibiting satisfactory sensitivity and specificity. This work opens a novel and easy route in designing advanced single-atom nanoprobe and expands their biosensing applications.

## Supplementary Information

Below is the link to the electronic supplementary material.

Supplementary file 1 (PDF 1248 kb)
