# Supplementary material for: Iron-Imprinted Single-Atomic Site Catalyst-Based Nanoprobe for Detection of Hydrogen Peroxide in Living Cells
Source: Nanomicro Lett. 2021 Jun 19;13:146. doi: 10.1007/s40820-021-00661-z (PMC8214641; doi:10.1007/s40820-021-00661-z)
Supplement: Supplementary file 1 — (PDF 1248 kb) [file 40820_2021_661_MOESM1_ESM.pdf]

Supporting Information for

Iron-Imprinted Single-Atomic Site Catalyst Based Nanoprobe for Detection of Hydrogen Peroxide in Living Cells

Zhaoyuan Lyu[1, †], Shichao Ding[1, †], Maoyu Wang[2], Xiaoqing Pan[3], Zhenxing Feng[2], Hangyu Tian[1], Chengzhou Zhu[1], Dan Du[1, *], Yuehe Lin[1, *]

[1]School of Mechanical and Materials Engineering, Washington State University, Pullman, WA, 99164, USA

[2]School of Chemical, Biological, and Environmental Engineering, Oregon State University, Corvallis, OR, 97331, USA

[3]Irvine Materials Research Institute (IMRI), University of California, Irvine, CA, 92697, USA

†Zhaoyuan Lyu and Shichao Ding contributed equally to this work.

*Corresponding authors. E-mail: yuehe.lin@wsu.edu (Yuehe Lin); annie.du@wsu.edu (Dan Du)

S1 Materials and Methods

S1.1 Materials

Tetraethyl orthosilicate (TEOS), [3-(2-Aminoethylamino)propyl]trimethoxysilane (A-Tri-EOS), 3,3′,5,5′-tetramethylbenzidine (TMB), 2,2′-azino-bis (3-ethylbenzothiazoline-6-sulfonic acid) (ABTS), and 1,2-diaminobenzene (OPD) were supplies by Sigma-Aldrich (St Louis, MO, USA). Sodium hydroxide and absolute ethyl alcohol were analytical grade and obtained from Fisher Scientific. Iron (III) nitrate was purchased from Alfa Aesar (Haverhill, MA, USA). All chemicals were directly used without further purification.

S1.2 Catalyst Characterization

The materials were characterized by transmission electron microscopy (TEM, Tecnai F20, 200 kV; Philips CM200 UT, 200 kV; JEOL ARM300F, 300 kV), X-ray photoelectron spectroscopy (XPS, Escalab 250, Al Kα), and X-ray diffraction (XRD, Rigaku Miniflex 600, 40 kV). X-ray absorption spectroscopy measurement at Fe K-edge was performed by Advanced Photon Source (APS) on the bending-magnet beamline 12-BM. The radiation was monochromatized by a Si (111) double-crystal monochromator. All spectra were collected in fluorescence mode by a germanium detector. XAS data reduction and analysis were processed by Athena software. The Fe content of the catalyst was evaluated with an Agilent inductively coupled plasma mass spectrometer (ICP-MS). Preparing a 5mg/ml sample aqua regia solution, the sample was gradually dissolved after 3days. Subsequently, the above

solution was diluted with nitric acid (3% v/v) and then subjected to the ICP-MS measurement.

S2 Experimental Section

S2.1 Peroxidase Activity and Kinetics Assay of Nanoprobe

The 3,3′,5,5′-tetramethylbenzidine (TMB), 2,2’-azino-bis(3-ethylbenzthiazoline-6-sulfonic acid) (ABTS) and o-phenylenediamine (OPD) were used as substrates for the colorimetric reaction of prepared samples. Moreover, TMB was further used as a typical substrate to study the peroxidase activity of the obtained single-atomic site catalysts (SASCs). Specifically, different amounts of IIM-Fe-SASC, NIM-Fe-SASC, and NIM were dissolved in 0.2 M pH 3.6 NaAc-HAc buffer, respectively. TMB was dissolved in DMSO to 10 mg mL^{-1} then added 100 μL to the above solutions. The obtained mixtures were incubated in dark at 37 ^{o}C for 5 min.

Then H_2O_2 solution was added to the above mixture to a final concentration of 1M, in which one sample was served as background measurement without adding H_2O_2. The reaction-time curve of IIM-Fe-SASC was plotted using the absorbance at 652 nm against the reaction time. The catalytic activity units (U) were evaluated by detecting the absorbance at 652 nm of a 10 s interval within 700 s. The catalytic activity expressed in units (U) was calculated through the following equation after eliminating background:

$$SA = \frac{V/(\varepsilon \times l) \times (\Delta A/\Delta t)}{m}$$

In which SA is the specific activity (U mg^{-1}); V is the volume of the reaction solution (μL); ε is the molar absorption coefficient of TMB substrate (39,000 M^{-1} cm^{-1} at 652 nm); l is the optical path length through reaction solution (cm); ΔA/Δt is the initial rate (within 1 min) of the absorbance change (min^{-1}) and m is the amount (mg) of SASCs in each assay.

The steady-state kinetic measurements of three peroxidase-like single-atom nanoprobes were measured according to the following steps. Firstly, 50 μL of 1 μg mL^{-1} samples in NaAc-HAc buffer (pH 3.6) solution was added to each well, various volumes of TMB solution (10 mg mL^{-1} in DMSO), then certain volumes of H_2O_2 solution were added to the reaction mixture to a concertation of 1M. The absorbance at 652 nm was immediately recorded at a 10 s interval within 60 s, the initial rates of the chromogenic reaction to different TMB concentrations were obtained and were fitted with Michaelis-Menten model. Furthermore, Michaelis constant K_m and K_{cat} were calculated according the following Michaelis-Menten equation:

$$v = \frac{v_{\max}[S]}{K_{\mathrm{m}} + [S]} \quad K_{cat} = \frac{v_{max}}{[E]}$$

where v is the initial rate of the chromogenic reaction, [S] is the TMB concentration and [E] is the concentration of catalysts (M).

To evaluate the temperature stability of the IIM-Fe-SASC, the samples were incubated at different temperatures for 2h, then conducted the experiments at room temperature. For determining pH stability of the IIM-Fe-SASC, we incubated samples at a higher concentration by HAc-NaAc buffers with different pH for 2h, then diluted the samples with pH 3.6 buffer, and then the peroxidase-like activity was tested under standard conditions.

S2.2 Cell Culture and Cytotoxicity Assay

MDA-MB-231 cells were cultured with Dulbecco's modified Eagle medium under oxidative stress conditions supplemented with fetal bovine serum (10%), and then kept in a humid environment with 5% CO_2 at a temperature of 37 ^{o}C. In vitro cytotoxicity of the material to MDA-MB-231 cells by standard 3-(4,5-dimethylthiazo-2-yl)-2,5-di-phenyltetrazoliumromide (MTT) analysis. The MDA-MB-231 cells were incubated in a 96-well plate at a cell density of 1.0×105/mL for 24 hours. The medium was then discarded and treated with Dulbecco's modified Eagle medium for 8 hours, which contained different concentrations of IIM-Fe-SASC nanoprobe or TMB. After that, the cells were incubated with 20 μL MTT (5 mg mL^{-1} in PBS) for 4 hrs. After the incubation process, 150 μL of DMSO was added to each well and then used a microplate reader to record the optical density at a wavelength of 490nm.

S3 Supplementary Figures

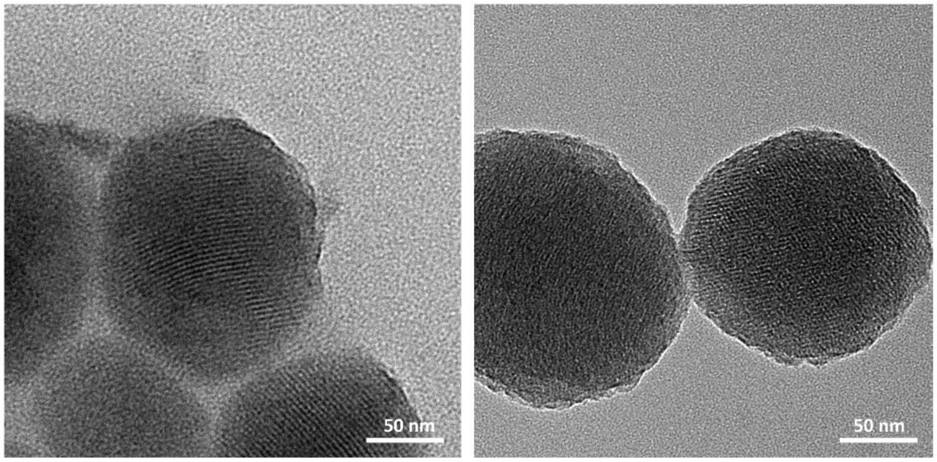

Fig. S1 TEM images of Fe-doped (left) and without doped (right) mesoporous SiO_2 precursor

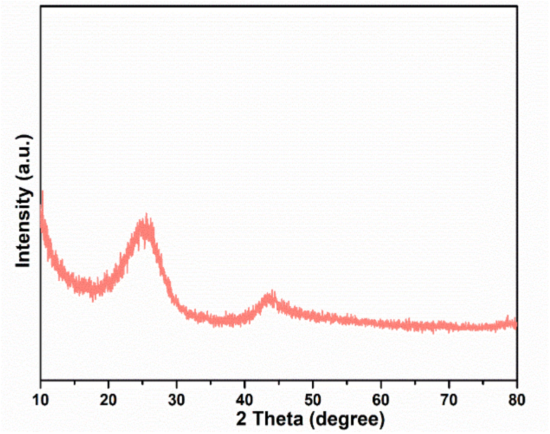

Fig. S2 XRD curves of IIM-Fe-SASC

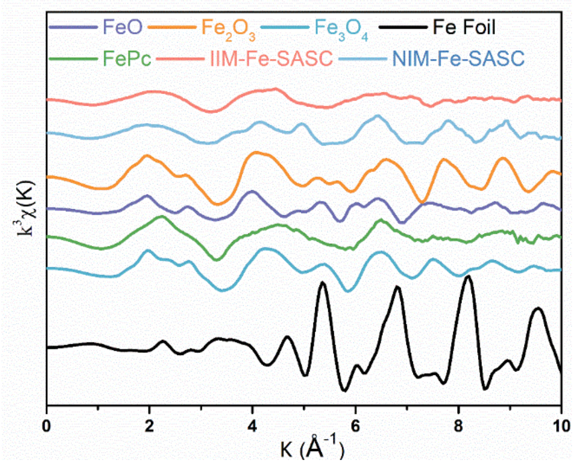

Fig. S3 K-edge EXAFS oscillations of IIM-Fe-SASC, NIM-Fe-SASC and reference samples of FePc, Fe foil, FeO, Fe_2O_3, and Fe_3O_4

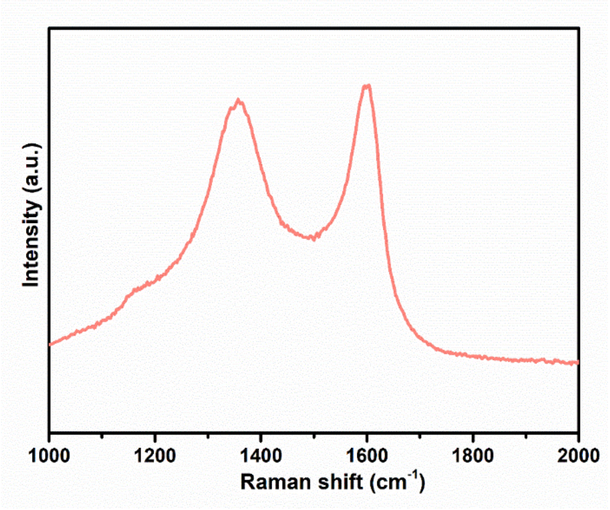

Fig. S4 Raman spectra of IIM-Fe-SASC

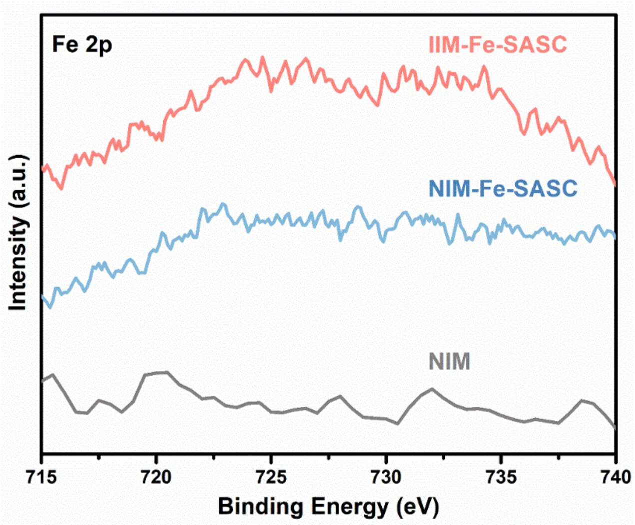

Fig. S5 Fe 2p XPS spectra of IIM-Fe-SASC, NIM-Fe-SASC, and NIM

Table S1 Comparison peroxidase-mimic activity of IIM-Fe-SASC, NIM-Fe-SASC, NIM, related reported nanozymes and HRP

Samples	Peroxidase-like specific activity (U/mg)	Refs.
IIM-Fe-SASC	48.5	This work
NIM-Fe-SASC	16.6	This work
NIM	4.4	This work
FeNC	4.09	Nano Today, 2020, 35, 100971
FeBNC	15.41	Nano Today, 2020, 35, 100971
SNC nanozymes	17.5	Analytical chemistry. 2020, 92, 13518.
Go/Fe-MOF	7.689	Analytical chemistry. 2019, 91, 13847.
Fe-MOF	5.086	Analytical chemistry, 2019, 91,13847.
Fe_3O_4 NPs	5.143	Nature protocols, 2018, 13, 1506.
Carbon NPs	3.302	Nature protocols, 2018, 13, 1506.
Au NPs	1.633	Nature protocols, 2018, 13, 1506.
Natural HRP	297	Biosensors and Bioelectronics, 2019, 142, 111495.

Table S2 Optimization of the $Fe(NO_3)_3$ amount in the precursor

Samples	Added $Fe(NO_3)_3$ amount (mg)	Fe loading amount (wt %)	Enzyme activity (U/mg)
IIM-Fe-SASC[1]	5	1.29	35.3
IIM-Fe-SASC	10	2.12	48.5
IIM-Fe-SASC[2]	15	2.81	38.6
NIM-Fe-SASC	10	1.41	16.6
NIM	0	0	4.4

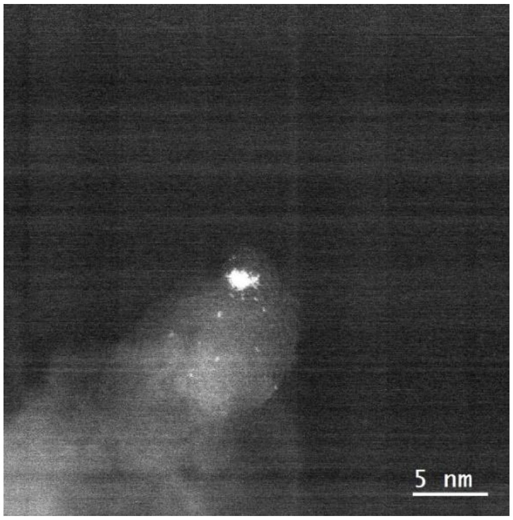

Fig. S6 HAADF-STEM images of IIM-Fe-SASC[2]

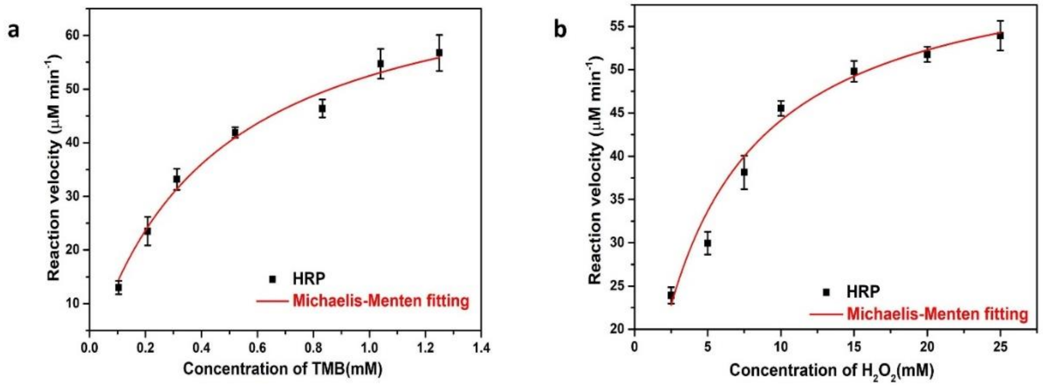

Fig. S7 Steady-state kinetics curves of HRP toward (**a**) H_2O_2 and (**b**) TMB

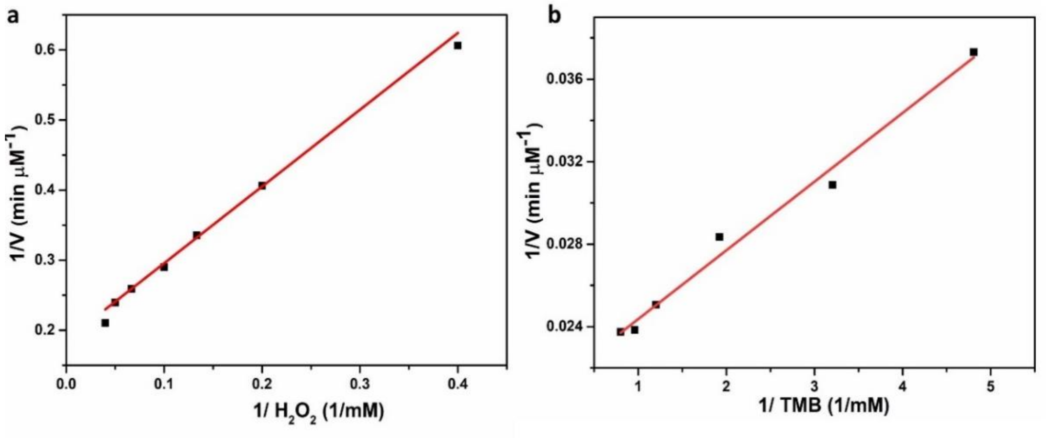

Fig. S8 Double-reciprocal plots of IIM-Fe-SASC for determining the kinetic constants for H_2O_2 and TMB substrate

Table S3 Comparison of steady-state kinetics parameters of IIM-Fe-SASC and the natural HRP

Materials	[*E*] (M)	Substrate	K_m (mM)	v_{max} (μM min^{-1})	K_{cat} (min^{-1})	K_{cat}/K_m (M^{-1} min^{-1})
IIM-Fe-SASC	2.23×10^{-11}	H_2O_2	6.63	5.65	2.49×10^5	3.7×10^7
		TMB	0.17	48.45	2.17×10^5	12.8×10^9
Natural HRP	5.2×10^{-11}	H_2O_2	4.52	64.08	1.23×10^6	27.2×10^7
		TMB	0.439	75.51	1.45×10^6	3.3×10^9

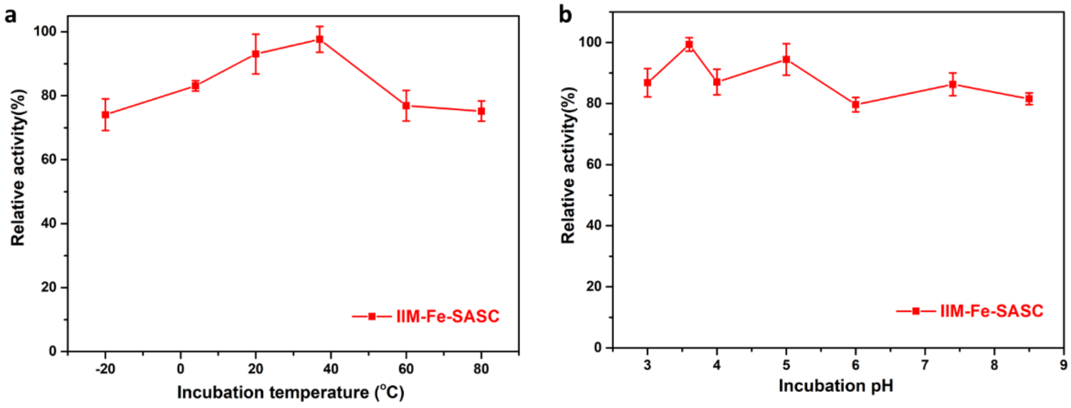

Fig. S9 Robustness of IIM-Fe-SASC against the harsh environment of temperature and pH

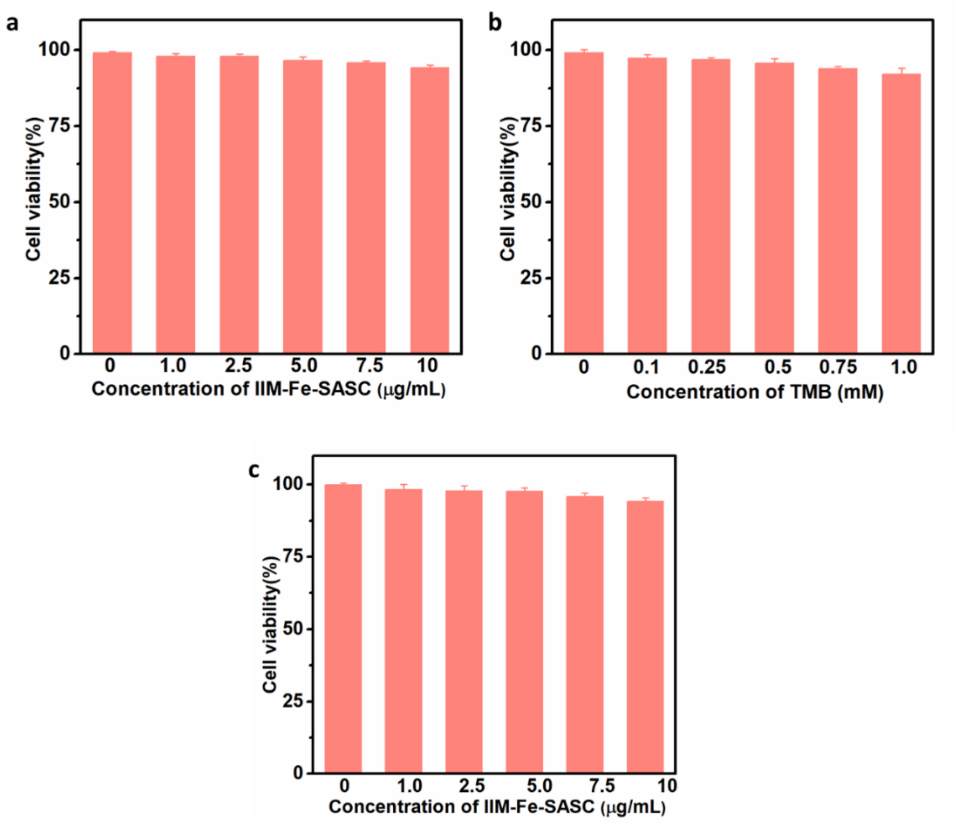

Fig. S10 MTT assay of IIM-Fe-SASC in MDA-MB-231 cells (**a**), TMB in MDA-MB-231 cells (**b**), and IIM-Fe-SASC in HBEC-5i cells (**c**)

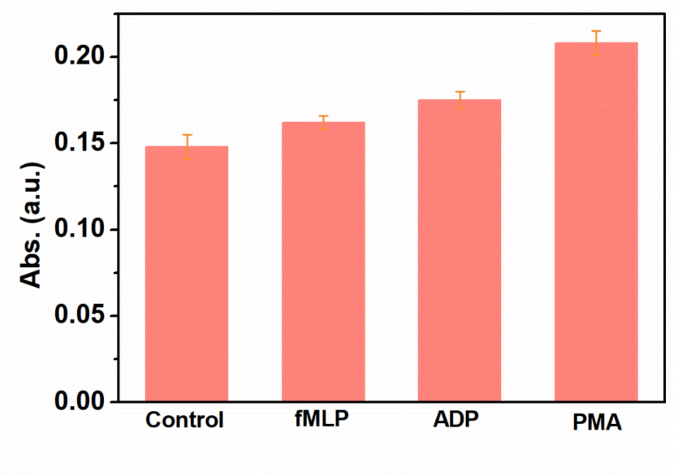

Fig. S11 Absorbance values of ox-TMB toward the fMLP, ADP, and PMA
